# Functional resonance magnetic imaging (fMRI) in adolescents with idiopathic musculoskeletal pain: a paradigm of experimental pain

**DOI:** 10.1186/s12969-017-0209-6

**Published:** 2017-11-14

**Authors:** Juliana Molina, Edson Amaro, Liana Guerra Sanches da Rocha, Liliana Jorge, Flavia Heloisa Santos, Claudio A. Len

**Affiliations:** 1 Researcher of Rheumatology Sector of Department of the Pediatrics, Escola Paulista de Medicina, Universidade Federal de São Paulo, São Paulo, Brazil; 2 Brain Institute and Department of Diagnostic Imaging Hospital Israelita Albert Einstein, São Paulo, Brazil; 3 Brain Institute, Hospital Israelita Albert Einstein, São Paulo, Brazil; 4 Hospital Israelita Albert Einstein, São Paulo, Brazil; 5 University of Minho, Braga, Portugal; 6 Pediatric Rheumatology Unit of the Department of Pediatrics, Escola Paulista de Medicina, Universidade Federal de São Paulo and Doctor of Department of Pediatrics, Hospital Israelita Albert Einstein, Rua Borges Lagoa 802 - Vila Clementino, São Paulo, SP CEP 04038-002 Brazil

**Keywords:** Musculoskeletal pain, Magnetic resonance imaging, Pain, Functional neuroimaging, Adolescents, Juvenile fibromyalgia

## Abstract

**Background:**

Studies on functional magnetic resonance imaging (fMRI) have shown that adults with musculoskeletal pain syndromes tolerate smaller amount of pressure (pain) as well as differences in brain activation patterns in areas related to pain.The objective of this study was to evaluate, through fMRI, the brain activation in adolescents with idiopathic musculoskeletal pain (IMP) while performing an experimental paradigm of pain.

**Methods:**

The study included 10 consecutive adolescents with idiopathic musculoskeletal pain (average age 16.3±1.0) and 10 healthy adolescents age-matched. fMRI exams were performed in a 3 T scanner (Magnetom Trio, Siemens) using an event-related design paradigm. Pressure stimuli were performed in the nondominant hand thumb, divided into two stages, fixed pain and variable pain.

The two local Research Ethics Committees (Ethics Committee from Universidade Federal de São Paulo- Brazil, process number 0688/11, on July 1st, 2011 and Ethics Committee from Hospital Israelita Albert Einsten – Brazil, process number 1673, on October 19th, 2011) approved the study.

**Results:**

The idiopathic musculoskeletal pain (IMP) group showed a reduced threshold for pain (3.7 kg/cm^2^ versus 4.45 kg/cm^2^, *p* = 0.005). Control group presented increased bain activation when compared to IMP group in the following areas: thalamus (*p* = 0.00001), precentral gyrus (*p* = 0.0004) and middle frontal gyrus (*p* = 0.03). In intragroup analysis, IMP group showed greater brain activation during the unpredictable stimuli of the variable pain stage, especially in the lingual gyrus (*p* = 0.0001), frontal lobe (*p* = 0.0001), temporal gyrus (*p* = 0.0001) and precentral gyrus (*p* = 0.03), when compared to predictable stimulus of fixed pain. The same intragroup analysis with the control group showed greater activation during the unpredictable stimuli in regions of the precentral gyrus (*p* = 0.0001), subcallosal area (*p* = 0.0001), right and left occipital fusiform gyrus (*p* = 0.0001; (*p* = 0.0007), middle gyrus (*p* = 0.01) and precuneus *p* = (0.02).

**Conclusion:**

Adolescents with idiopathic musculoskeletal pain (IMP) tend to request higher brain function in cognitive-emotional areas when interpreting unpredictable sensory-perceptual situations. Therefore, it is assumed that this difference in pain processing in adolescents with IMP make the subjective experience of pain something more intense and unpleasant.

## Background

According to criterias established by Malleson in 1992 [1], Idiopathic musculoskeletal pain (IMP) is defined by the occurrence of intermittent and generalized musculoskeletal pain in three or more spots for at least 3 months, excluding other diseases, for example rheumatic, neoplastic and infectious diseases, which may justify the pain complaint [1]. It affects around 12% to 35% of children and adolescents in school age [2–8]. Common in girls, the symptoms usually starts around 12–13 years and has its incidence peak at around 14 years [7, 8], with significant impact and damage in social, school and family of patients [5, 9, 10]. It is known that constitutional and environmental factors play an important role in triggering the IMP. There are a few hypotheses about the intrinsic factors related to IMP, which includes aspects of nociception and factors related to the pain threshold, in this case a differentiation in the the pain-related activation areas in the brain [11–13].

Since 1968, the basic understanding about pain comes from the theory proposed by Melzack and Casey [14], where the pain processing occurs through sensory and emotional components processed in parallel by different brain structures. Therefore, sensory-discriminative aspects, such as type, location and intensity, are processed by areas such as lateral thalamus, somatosensory cortex 1 and 2 (S1 and S2) and posterior parietal cortex, while affective-motivational aspects are processed by areas such as thalamus lateral, prefrontal cortex and limbic system [14–17]. After some additional studies, cingulate became part of this network [18].

In an attempt to expand the research about the brain mechanisms of pain processing in recent years, studies have been conducted with the aid of refined neuroimaging techniques and paradigms of experimental pain in patients with musculoskeletal pain syndromes, such as fibromyalgia and complex regional pain [11, 16, 19–26]. These studies, performed using functional magnetic resonance imaging (fMRI), demonstrated that adult patients with fibromyalgia (FM), a subclassification of IMP syndromes, tolerate a smaller amount of pressure (pain) and showed differences in brain activation patterns in cortical and subcortical areas related to pain, especially in the cortex of the cingulate, insula, S1 and S2, as well as brain volume changes, when compared to healthy controls, i.e. without complaints of chronic pain [11, 27, 28]. Studies with fMRI in adults that evaluated the aging effect on the brain showed changes in the pattern of gray and white matter in accordance with the age of patients with fibromyalgia, as well as a strong correlation between smaller amount of gray and white matter with greater sensitivity to pain [21]. Lebel et al. suggest that changes resulting from chronic pain occurring at a time of intense development and neuroplasticity may modify the pain processing mode in adolescents with complex regional syndrome [13]. However, there is no data on when these changes get started and few studies involving children and adolescents with IMP.

The main objective of this pioneer study was to evaluate, using fMRI techniques, the brain activation in adolescents with IMP during an experimental paradigm of pain. As hypothesis, different patterns of brain activation are expected in areas related to pain matrix (primary and secondary somatosensory, insular, anterior cingulate, and prefrontal cortices and thalamus) [29–31].

## Methods

Model: Descriptive, cross-sectional case-control study.

### Ethical aspects

The two local Research Ethics Committees (Ethics Committee from Universidade Federal de São Paulo- Brazil, process number 0688/11, on July 1st, 2011 and Ethics Committee from Hospital Israelita Albert Einsten – Brazil, process number 1673, on October 19th, 2011) approved the study. All participants and their guardians were informed about the procedures, risks and conducts. The fMRI was performed after the signature of the consent form. All participants and their guardians agreed to the study and future publications of the results.

### Participants

From a population of 74 IMP patients (aged between 5 and 18 years old) followed in our pediatric musculoskeletal pain clinic, 12 adolescents were consecutively selected according to the criteria of Malleson et al. 1992 [1]. All patients were under medical treatment by a pediatric rheumatologist for at least 6 months. There were excluded patients with following clinical aspects: history of psychiatric and/or neurological disorders diagnosticated by neurologist or history of, traumatic brain injury; use of drug or psychoactive drugs; incidental findings and changes in neural structural images, such as tumors and cysts; patients with fMRI contraindications conditions, such as use of metal clips, implants, braces or unremovable piercings.

The fMRI exam was then applied to 12 adolescents with IMP, aged between 14 and 17 years, of both genders and 11 matched controls, without history of pain, tanner stage 5 [32], selected in the same social groups (school and neighborhood) through indications made by adolescents with IMPTwo subjects from IMP group were excluded, one due to use of braces and another due diagnosis of Turner syndrome.

Both participants of the IMP group, during the recruitment telephone contact did not report the existence of conditions incompatible with the selection criteria (hidden dental braces and the diagnosis of Turner syndrome). These conditions were only observed when the adolescents were already in the research institute and, finally, and 1 adolescent from the control group due to incidental finding of a cyst. The final sample consisted of 20 adolescents, 10 allocated in IMP group (9 girls and 1 boy, average age 16.3 ± 1.1) and 10 healthy adolescents in the control group (9 girls and 1 boy, mean age 16.1 ± 1.4) with no complaint of pain.

It is worth mentioning that all adolescents of the IMP group also had positive diagnosis for juvenile fibromyalgia, according to the American College of Rheumatology criteria [33, 34].

None of the adolescents were under continuous use of pain medication, yet they were all advised not to use any pain medications (including prescription-free) during the last 24 h before the test.

### Procedures

#### Sample characterization

The sample was characterized as the following questionnaires: A) *Pediatric Quality of Life Inventory - Version 4.0 (PedsQL 4.0) Brazilian adaptation for free use* [35] - measurement of children’s quality of life and healthy adolescents and patients with chronic diseases, parents and tens Version; B) *Stress Scale in Adolescents – ESA* [36] - evaluation of symptoms related to stress reactions; C) *Beck Depression Inventory - BDI-II* [37] - evaluation of symptoms corresponding to DSM-IV criteria for diagnosing depressive disorders; D) *Visual analogue scale – VAS* [38] - quantification of pain intensity.

#### Pain paradigm

Discrete pressure stimuli, lasting 2 s, were applied to the nondominant thumb by means of hard rubber tube connected to a hydraulic piston, which were connected by a combination of a second piston valve. This second piston transmitted the pressure of standardized weights laid out on a platform for controlled and reproducible pressure stimuli. The experimental pain equipment is designed exclusively for the study of fMRI [39].

**Fig. 1:**
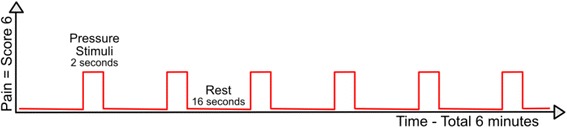
Experimental design of the fixed pain

**Fig. 2:**
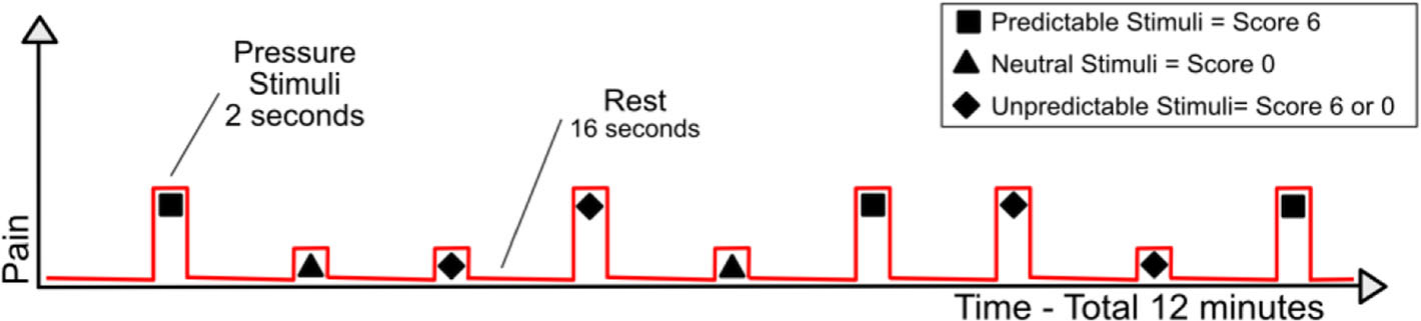
Experimental design of the variable pain

It was used a paradigm of event-related design, divided into two steps: 1) **Fixed Pain**, in which for 6 min participant received 22 pressure stimuli of pain subjective to score 6 (six) with interval of 16 s, preceded by display of a cross symbol on the screen, as shown in Fig. 1; 2) **Variable pain**, in which for 12 min participant received 45 randomly distributed pressure stimuli were applied every 16 s, however with 2 different intensity possibilities (0.5 kg/cm^2^ or equivalent pressure to score 6) in 3 conditions: A) *predictable pain stimuli*, when the participant visualize a square (■) on the screen and then received the pressure stimulus with intensity equivalent to score 6 of pain; B) *unpredictable pain stimuli*, when participants visualized a diamond (◆), then, could receive either a pressure stimulus score 0 (zero) or score 6 (six) in order to not be able to predict what stimulus would be administered. C) *neutral stimuli*, a triangle (Δ) was presented on the screen and then received the pressure stimulation with intensity of 0.5 kg/cm^2^, corresponding to score 0 (zero) pain, as shown in Fig. 2.

The same protocol was applied to all participants, with all steps described above. They were not previously informed about the meaning of each symbol visualized prior to the stimuli (ie, which symbol would be followed by pain, non-pain, and unpredictable stimuli).

#### Calibration of pressure stimuli

The intensity of the stimuli was previously calibrated for each participant. Each participant was asked to verbally graduate the intensity of the pain felt for each stimulus by a numeral analog scale of 0 to 10. The session consisted of an ascending series of pressure stimuli, starting with 0.5 kg/cm^2^ and increasing 0.5 kg/cm^2^ to the limit of tolerance or up to 9.0 kg/cm^2^.

The stimuli were applied twice in ascending order and, within a short break, applied a third time in descending order to confirm the degree of pressure. For each participant, the pain threshold was defined as the greatest stimulus, whose intensity received at least two equal scores, being assigned to it, score 6 (six) of pain on a scale of 0 to 10. Our study followed the same parameters and methodology adopted by international studies [11, 20, 22–26, 40].

#### fMRI instrumentation

All tests were performed in a 3.0 T Magnetom Trio equipment: TIM System (Siemens Medical - Germany) with 40 mT/m gradients, increase ratio of 230 mT/m/s and dedicated receptive coil of 12 elements.

Functional images were acquired by sequence of T2 echo planar image (EPI-BOLD) for the whole brain, capturing the signal contrast variations according to the *Blood Oxygenation Level Dependent* (BOLD) effect with the following conditions: - GRE EPI T2 - BOLD: TR = 2000 ms, TE = 30 ms, thickness of 5 mm, 0.5 mm range, FOV 200 mm and matrix of 64 × 64, with 405 volumes, neglecting the first 4 TR’s, for the decrement signal.

BOLD contrast is the MR imaging method most often used to produce information related to brain function.

This method is based on MR images made sensitive to changes in the state of oxygenation of the hemoglobin [41]. This molecule has diVerent magnetic properties depending on the concentration of O2; when it is fully saturated with oxygen (oxyhemoglobin) it behaves as a diamagnetic substance, while when some oxygen atoms have been removed (deoxyhemoglobin) it becomes paramagnetic.

Within any particular imaging voxel (representing a small part of the brain) the proportion of deoxyhemoglobin relativeto oxyhemoglobin dictates how the MR signal will behave in a BOLD image: areas with high concentration of oxyhemoglobin give a higher signal (a brighter image) than areas with low concentration [42].

For stimulus presentation and acquisition of behavioral responses was used the NNL system (NordicNeuroLab Inc., Norway) via dedicated algorithm (E-prime - *Psychology Software Tools, Inc., USA*). This system uses independent binocular projection via LCD screen and hand response detection through keyboard compatible with magnetic environment.

### Data analysis

All behavioral data were exposed as **average ± standard deviation**. The variables were compared between groups using the Student-t test for independent samples in the Statistica Software (Statsoft). The significance level of *p* < 0.05 was adopted.

The obtained functional images were processed and analyzed by the statistical program FSL version 4.1 (FMRIB Software Library - Analysis Group, Oxford, UK - http://www.fmrib.ox.ac.uk/fsl/), specifically the FEAT module (FMRI Expert Analysis Tool) version 5.98 (to detect brain activation based on changes in the BOLD signal).

The data were processed in three steps: pre-processing, statistical analysis and presentation of the activation images. Individual activation maps were obtained using the general linear model (GLM).

The images obtained from the statistical analysis with Student-t test indicated the regions where the signal varied significantly when comparing to the periods of activation and rest. It was calculated, for each pixel, the activation coefficient and the corresponding z-score. Analyzing the BOLD signal corresponding to the fMRI signal difference between the activation states and rest, the z-score is calculated taking into account the average value (μ) and standard deviation (σ).

A minimum statistical threshold of 1.9 (*p* < 0.05) was used to determine which voxels have been activated during the pain paradigm execution. The anatomical regions were identified by overlapping those on structural images. The maps of the atlas system MNI 152 (Montreal Neurological Institute) and Harvard-Oxford, available in FSLview software, were used for the classification of the active areas.

## Results

Table 1 presents demographic data, years of schooling, PedsQL 4.0, SSA, BDI, and pain VAS for idiopathic musculoskeletar pain syndrome patients and controls. In the total sample, 3 participants were left-handed (1 in IMP group and 2 in the control group). Covariance of this variable was considered on fMRI analysis.

**Table 1:** Demographic data, years of schooling, PedsQL, SSA, BDI-II, and VAS for idiopathic musculoskeletar pain syndrome patients and controls

	IMP	Controls	*p* Value
N=	10	10	
Age	16.3 ± 1.09	16.1 ± 1.3	0.36
Years of schooling	10 ± 1.5	9.6 ± 1.6	0.28
PedsQL Parents	1205 ± 491	1935 ± 473	0.003
PedsQL Adolescents	1470 ± 399	1872 ± 246	0.007
SSA	2.4 ± 0.7	1.9 ± 0.8	0.08
BDI-II	12.4 ± 11.6	7.5 ± 8.9	0.15
Pain score pre examination – VAS	3.2 ± 0.8	0	<0.0001^a^
Weight used (= grade 6/10)	3.7 kg/cm^2^	4.45kh/cm^2^	0.005

*PedsQL parents* Pediatric Quality of Life Inventory - Version answered by parents, *PedsQL Adolescents* Pediatric Quality of Life Inventory - Version answered by adolescents, *SSA* Stressscale for adolescentss minimum required score = 3.11, *BDI-II* Beck Depression Inventory, minimum required score = 13, *VAS* visual analogue scale

^a^Statistical Valor *p* = 0.000000000008

The PedsQL 4.0 scores related to health showed differences between the groups, from both the points of view, adolescents (*p* = 0.007) and their parents/guardians (*p* = 0.003), where the group of adolescents with IMP showed lower scores. The IMP patients also reported complaints of spontaneous pain, with an average score of 3/10 (VAS) in the pre-exam time.

From a clinical point of view, based on the results obtained on the scales, it is possible to say that our sample showed no differences in the occurrence of stress-related symptoms (SSA: 2.2 ± 0.8; *p* = 0.08), nor indicative symptoms of depression (BDI-II: 10 ± 10.4; *p* = 0.15). Means and standard deviations of the sample characterization are shown in Table 1.

### Intergroup analysis

#### Weight used

The adolescents of the IMP group demonstrated a significantly reduced pain threshold when compared to healthy adolescent group (3.7 kg/cm^2^ versus 4.45 kg/cm^2^, *p* = 0.005). By the end of the experiment, the average pain score reported by the participants for this pressure stimuli in the fMRI paradigm was 7.7 ± 1.7, on a scale of 0 to 10, varying from the initial score of 6.

#### Fixed pain

**Fig. 3:**
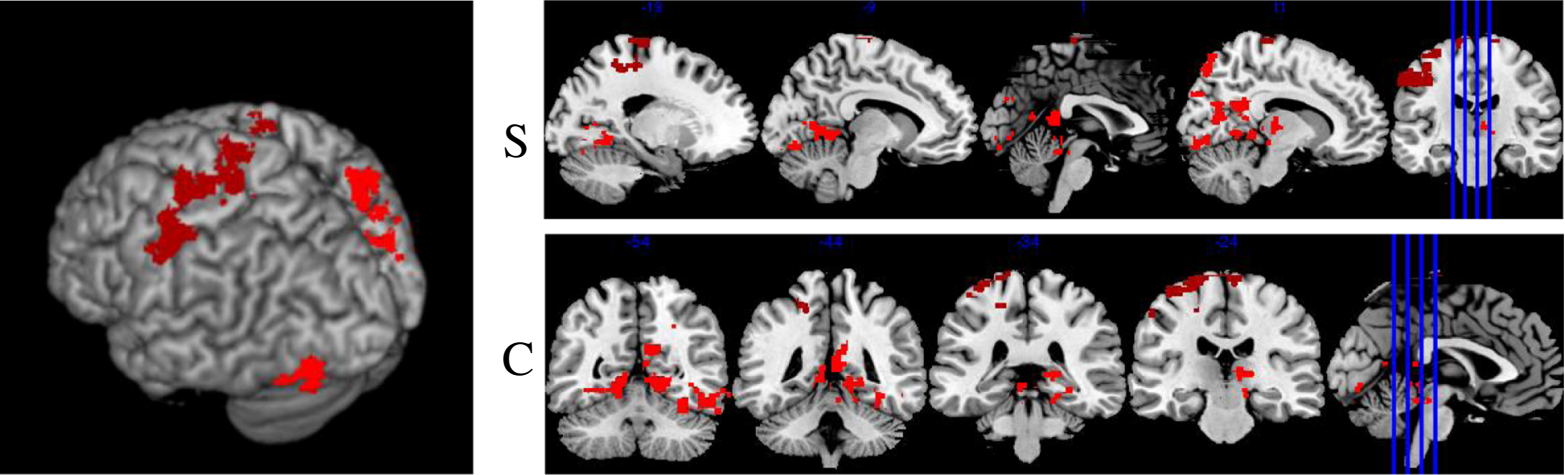
3D map view sagittal (**s**) and coronal (**c**) cuts the difference in activation during fixed pain paradigm in controls

The comparison between the two groups during execution of fixed pain paradigm, i.e. maximum calibrated pressure to each individual, equivalent to subjective pain equal to 6 (IMP group, 3.7 kg/cm^2^ ± 0.483 kg/cm^2^; Controls, 4.45 kg/cm^2^ ± 0.685 kg/cm^2^), showed increased brain activation in the control group, as shown in Fig. 3, when compared to the IMP group in 3 clusters: the thalamus, precentral gyrus and middle frontal gyrus. Table 2 describes the main anatomical sections of greater activation according to the MNI coordinates. It is important to emphasize that the IMP group activation maps did not show increased activation during the fixed pain paradigm.

**Table 2:** Description of clusters and significantly greater activation spots during the fixed pain paradigm in the control group

			MNI Coordinates^b^				
Localization	Side	Cluster size	x	y	z	Z score	*p* Value
1. Thalamus	R	5414	18	−28	4	3,07	<0.0001^a^
*Extending to*							
Parietal Lobe	R		30	−76	34	3,05	
Lingual Gyrus	R		18	−40	−12	3,02	
Inferior temporal gyrus	R		50	−52	−16	2,85	
2. Precentral gyrus	L	1643	−16	−20	78	3,38	0.0004
*Extending to*							
Poscentral Gyrus	L		−60	−14	42	3,19	
Precentral gyrus	R		10	−30	78	3,16	
3. Middle frontal gyrus	R	878					0.03
*Extending to*							
Inferior frontal gyrus	R		40	34	14	3	

^a^Statistical Valor *p* = 0.000000000008

^b^The presented coordinates refer to the location of the maximum activation voxel within each cluster. Statistical value *p* = 7e-12 (scientific notation for 0.000000000007)

### Intragroup analysis

#### Fixed pain x variable pain

**Fig. 4:**
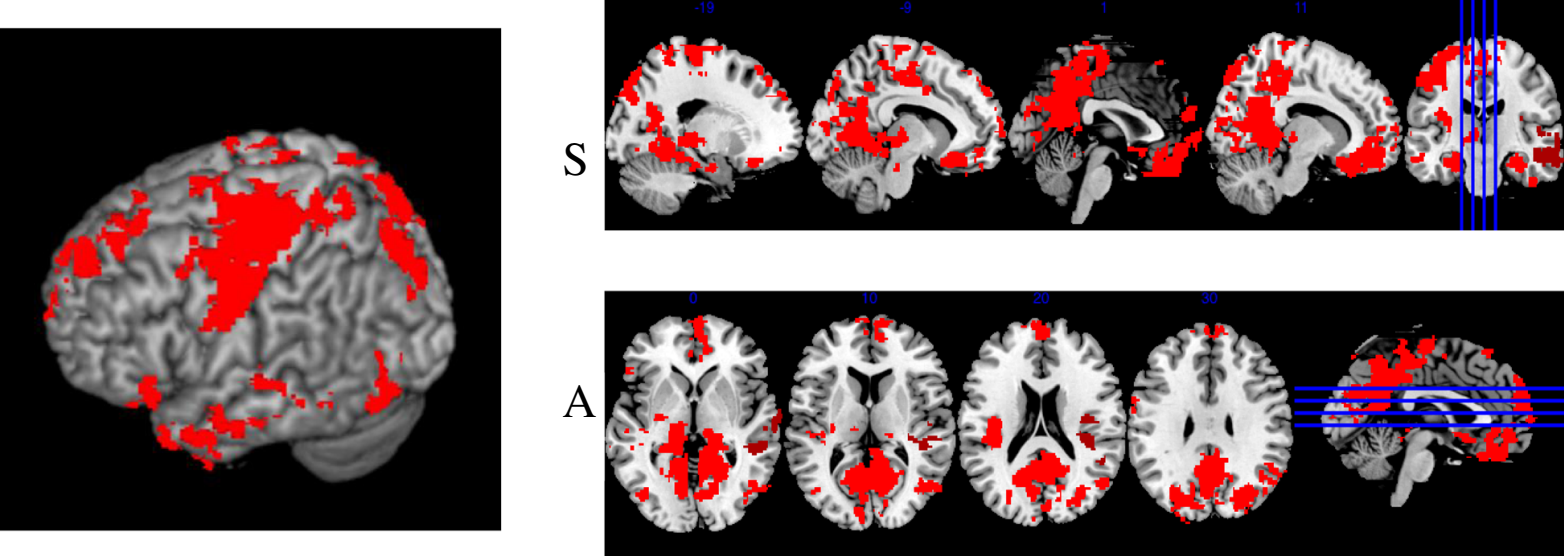
3D map view and sagittal (**s**) and axial (**a**) cuts of the difference in activation between the unpredictable stimuli of variable pain versus fixed pain stimuli in IMP group

The comparison between the fixed pain paradigm versus the moments of unexpected pain of variable pain paradigm, considering in the latter only the moments in which the pressure of score 6 was applied, showed in IMP group four greater activation cluster during the unpredictable pain, all of which are in the right hemisphere: lingual gyrus, prefrontal cortex, inferior temporal gyrus and precentral gyrus, shown in Fig. 4 and described in Table 3. In this table are also described the main areas and location of the activated voxels within these four clusters, while presenting the pressure stimuli.

**Table 3:** Description of significantly higher activation clusters during unpredictable variable pain IMP group when compared to fixed pain

			MNI Coordinates^a^				
Localization	Side	Cluster Size	x	y	z	Z score	*p* Value
1. Lingual Gyrus	R	21,632	8	−52	2	3.86	<0.0001^b^
*Extending to*							
Precuneus Gyrus	R		6	−56	8	3,63	
Cingulate gyrus	R		4	−50	28	3,55	
2. Prefrontal Cortex	R	4654	2	−42	−24	3,37	<0.0001^c^
*Extending to*							
Subcallosal area	R		6	30	−24	3,32	
Paracingulate Gyrus	R		4	50	−8	3,2	
3. Inferior temporal gyrus	R	2626	46	10	−42	3,29	<0.0001^d^
*Extending to*							
Middle temporal gyrus –posterior	R		56	−16	−16	3,04	
Superior temporal gyrus –posterior	R		68	−10	−4	2,93	
Planum temporale	R		42	−36	16	2,91	0.03
4. Precentral Gyrus	R	866	48	−2	22	2,66	
*Extending to*							
Poscentral Gyrus	R		66	−8	30	2,43	

^a^The presented coordinates refer to the location of the maximum activation voxel within each cluster. ^b^Statistical value *p* = 5.5e-30; ^c^Statistical value *p* = 8.19e-10 ^d^statistical value *p* = 3.16e-06 (idem scientific notation Table 2)

**Fig. 5:**
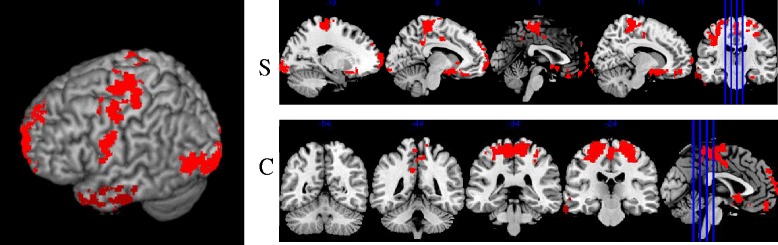
3D map view and sagittal (**s**) and coronal (**c**) cuts of the differences in activation between the unpredictable stimuli of variable versus fixed pain pain stimuli in the control group

In contrast, when the unpredictable painful stimuli were administered, the control group showed activation in 6 clusters distributed in both hemispheres, located in regions of the precentral gyrus, subcallosal area, left and right fusiform occipital gyrus, middle gyrus and precuneos. This data is presented in Fig. 5 and described in Table 4.

**Table 4:** Description of significantly higher activation clusters during unpredictable variable pain in the control group when compared to fixed pain

			MNI Coordinates				
Localization	Side	Cluster size	x	y	z	Z score	*p* Value
1.Precentral gyrus	R	4923	22	−24	68	3.25	<0.0001^a^
*Extending to*							
Poscentral Gyrus	R		12	−36	64	3.21	
Precentral Gyrus	L		−28	−26	62	3.13	
2. Subcallosal area	R	2833	10	10	−16	3.06	<0.0001^b^
*Extending to*							
Subcallosal area	L		−8	4	−16	3.02	
Middle frontal gyrus	L		−18	70	10	2.87	
3. Fusiform occipital gyrus	R	1366	36	−92	−10	3.04	0.0001
*Extending to*							
Middle temporal gyrus			46	−70	0	2.85	
4. Fusiform occipital gyrus	L	1120	−26	−94	−12	3.03	0.0007
5. Middle temporal gyrus	L	717	−64	−20	−24	2.99	0.01
6. Precuneus	R	667	24	−78	44	2.54	0.02

The presented coordinates refer to the location of the maximum activation voxel within each cluster. ^a^Statistical value *p* = 3.6e-13; ^b^statistical value *p* = 1.32e-08;

## Discussion

We observed differences in the brain activation pattern during pain, when comparing adolescents from IMP and healthy groups. The brain activation results pointed to areas mainly related to the pain affective-motivational domain. To our knowledge, this is the first fMRI study about the brain function using an experimental paradigm of pain in a group of adolescents with IMP.

In addition to imaging studies, other relevant issues on IMP were evaluated, such as quality of life and the occurrence of indicative symptoms of stress and depression.

Since our sample included a population in age from full development and brain plasticity, we took care to pair the factors with possible influences on brain development, such as age and educational level.

In our clinical practice, IMP adolescents and their families come to clinic visits with many complaints about social and emotional difficulties in their day-to-day. The poor PedsQL 4.0 scores showed the perception of adolescents with IMP. As expected, we observed worst scores in physical, educational, emotional and social dimensions of patients.

Also, behavioral data indicates similarities in relation to emotional aspects in both groups. The occurrence of stress and depression disorders in both groups was not identified.

Depression is highlighted as one of the most common symptoms in adult patients with IMP, especially in fibromyalgia [33, 43], still being related to differences in brain processing of pain in these patients [19, 20, 44, 45].

Unlike the findings in adults, but corroborating previous studies [46] for the pediatric population, our negative results for symptoms of stress or depression suggest that, despite a number of complaints and functional impairment, adolescents with IMP do not seem to yet experience significant mood changes found in adults with chronic pain.

The search for factors related to pain pathogenesis is in a fruitful field, because little is known about the subject. Recent studies show that the uses of matched experimental paradigms to sophisticated neuroimaging techniques allow a better understanding of brain mechanisms of pain processing. Pain is a complex and subjective experience recruiting the operation of multiple and different brain areas, including the primary and secondary somatosensory cortex, thalamus, prefrontal cortex, cingulate gyrus and insula [29].

However, cerebral pain processing is not limited only to these locations, since simple stimulus like sting of a needle or pressure on the skin can lead to activation of a larger number of brain areas located in cortical and subcortical areas. Studies on the assessment of brain response through paradigms of experimental pain in patients with chronic pain suggests that greater sensitivity to stimuli reported by these patients is accompanied by increased brain responses [11, 26].

The relevance of the research of brain processing during pain also in the pediatric population should be the search for better outcomes and behaviors in order to minimize long-term damage caused by chronic pain. Lebel et al., in the only study with fMRI in pediatric patients with complex regional pain syndrome, suggest that due to chronic pain changes in the central nervous system that occur in this stage of development and neuroplasticity may alter the pain processing on these individuals during their lifes [13].

During calibration of pressure stimuli, adolescents with IMP had a lower pain threshold than their healthy peers, similar to what happens in adults with pain idiopathic syndromes [19, 20].

In the fixed pain paradigm used in our study, all presented pressure stimuli had the same intensity, at constant intervals. In this paradigm, the control group showed greater brain activation compared to the group with IMP, and these activations were in areas related to sensory perception, motor perception and pain perception, such as thalamus, S1 and S2. Unlike most studies with adults [11, 20, 22, 26], our results suggest a greater activation of areas only in healthy adolescents, i.e. areas related to sensory-discriminative aspects of pain. However, we must highlight the fact that all adolescents IMP group reported some kind of spontaneous pain, with grades ranging from 2 to 4 in the VAS scale (0–10), since even before the administration of pressure tests, a fact absolutely opposite to the control group. This is not mentioned in studies with adults [11, 22, 23, 26].

This finding is relevant, especially when we analyze the results of the next stage, when the unpredictability factor was added to variable pain paradigm. When exposed to unpredictable pain stimuli, both groups showed greater brain activation when compared to fixed paradigm pain stimuli.

In IMP group, the main differences occurred in areas related to processing information related to emotional aspects, such as self-awareness and emotional regulation (precuneos, cingulate gyrus, subcallosal area) and also in areas involved in the interpretation of sensory stimuli, as images and somesthesic information and motor (temporal gyrus, pre and poscentral gyrus). In contrast, in the control group, the main activation clusters were in areas involved in the interpretation of sensory stimuli, such as image processing, somesthesic information and motor (temporal gyrus, pre and poscentral gyrus, fusiform gyrus and middle temporal gyrus) and you can also identify clusters in areas emotional regulation (precuneos and subcallosal area).

Thus, in general, both groups showed an increase in the BOLD effect during the unpredictable pressure stimuli compared to fixed pressure stimuli. However, it is possible to notice a difference in the pattern of this increase in activation, especially in IMP group, where the main areas of activation related to the affective-motivational system, for example, the cortex of the cingulate gyrus and prefrontal cortex.

It is possible to observe the role of the cingulate gyrus to process unpleasant aspects of pain, such as the integration of these sensations with affection, cognition and selection behavioral response [47]. Since the role of the prefrontal cortex is associated with better motivational and anticipatory aspects of pain [48]. Cognitive and affective factors, such as attention, anticipation and anxiety, can also be understood as aspects not related to pain. However, these factors can influence the modulation of pain perception due to the fact of pain processing is also closely related to brain areas related to these aspects [48]. It is important to consider that our experimental paradigm was not specifically designed to detect involvement of anxiety and anticipation in pain, as participants were previously instructed on the meanings of the symbols that preceded the pressure stimuli, although they could deduce over time. The purpose of this design was to investigate the impact of unpredictability and participation of involuntary attention in pain processing. In the study of Petzke et al. [49], thermal and pressure, stimuli with different intensities presented randomly caused increased brain activation in both adult controls as in adult patients with fibromyalgia, when compared to stimuli presented in ascending order.

As already shown in some studies with adults [20, 27, 50], adolescents in our study demonstrate significantly increased BOLD effect in regions involved in emotional/cognitive aspects related to pain processing, reinforcing the hypothesis that affective/emotional aspects attributed to the frontal-cingulate regions that have a relevant role on pain processing also in adolescents with IMP.

Perhaps these differences in processing are indicative factors that lead some people to experience more pain.This circuit frontal-cingulate seems to be a key component in the process of understanding how the pain processing occur in adolescents with IMP, since it is involved in functional executive systems as well as pain-related systems. In the lights of our findings, we can assume that these adolescents require more brain effort in cognitive-emotional areas when interpreting unpredictable sensory-perceptual event..

In our study, some factors associated with pain within menstruation cycle, time with pain and type of therapeutic treatment (medical, psychological, physiotherapy among others) were not considered in the analysis. The sample size is small, even if we take into account the socioeconomic, educational and emotional aspects have been considered.

## Conclusion

In front of new or conflicting situations, our results suggests that adolescents IMP tend to request higher brain function in order to interpret them. In addition, these regions, known to be also involved in attentional and executive processes (mainly in the selection of responses and/or conflict resolution) are also required in the processing of pain. Therefore, it is assumed that this difference in pain processing in adolescents with IMP make the subjective experience of pain something more intense and unpleasant. Our results emphasize the importance of early diagnostics and constant therapeutic monitoring as these may prevent the occurrence of mood disorders, common in adults with IMP.
